# What Patients with Bipolar Disorder Need to Know about Lithium

**DOI:** 10.3390/ph17091223

**Published:** 2024-09-17

**Authors:** Robert M. Post, Janusz K. Rybakowski

**Affiliations:** 1 Bipolar Collaborative Network, Chevy Chase, MD 20815, USA; 2 Department of Adult Psychiatry, Poznan University of Medical Sciences, 61-701 Poznań, Poland; janusz.rybakowski@gmail.com

**Keywords:** lithium, bipolar disorder, therapeutics

## Abstract

Lithium is the superior first-line treatment for bipolar disorder (BD). Yet the percentage of patients receiving lithium is abysmally low, especially in the US. Since psychiatrists have failed to place lithium in its appropriate role, we make the case that patients with BD themselves need to be better educated about the unique characteristics and pre-eminence of the drug so that it can be used more often and appropriately. Lithium has a highly unfavorable popular reputation among would-be patients and many psychiatrists. Thus, a direct appeal to patients with BD appears appropriate to try to remediate this situation. The unique assets of lithium are underappreciated or not well known. Conversely, the side effects profile of lithium are overestimated. Here, we make the case that lithium’s image needs to be revised not only with better and more accurate information but also with a wholesale renaming and rebranding of the drug. We will not only outline the unique qualities and new information about the side effects of the drug but attempt to change some of the terminology conventionally used to refer to lithium so that its use may be appropriately applied earlier and at an increased frequency for patients with BD.

## 1. Introduction

Lithium is the acknowledged superior first-line treatment for bipolar disorder [1]. Yet the percentage of patients receiving lithium is abysmally low, especially in the United States. Baldessarini et al. [2] found that the first class of drugs prescribed for bipolar patients was antidepressants in 50% and lithium in only 8%. Rhee et al. [3] found that in the last decade and a half, the use of antipsychotics increased from 12.4% to 51.4%, while the use of lithium decreased from 30.4% to 17.6%. Besides the US, the decline of lithium use in recent years was observed in Europe but not in Asia [4]. Elsewhere, we have argued that lithium should be reconsidered not only as a first-line drug, but also as a potential disease-modifying drug (DMD) [5], and as such, it should be used earlier and more often to reduce disability and dysfunction.

Lithium is unparalleled in its ability to prevent episodes of mania and depression [6,7]. It also has the best data for preventing suicides. It is most effective when initiated early in the course of bipolar disorder, and when used in this fashion has a major ameliorative effect on the series of adversities that are directly linked to the experience of increasing numbers of episodes [8]. These include an increased frequency and more rapid rate of recurrences; greater severity of recurrences; and episodes requiring fewer precipitating stressors. These sensitization and kindling-like processes emphasize the importance of early and sustained use of the best agents for preventing episodes. The underutilization of lithium is particularly problematic in the United States (US) where there is a higher incidence of childhood-onset bipolar disorder, and one-quarter of bipolar disorder in well-diagnosed adults begins before age 13 and two-thirds begins before age 19 [9,10,11]. These earlier onsets in the US are associated with greater illness severity as manifested by more childhood stressors; more anxiety disorders and substance abuse comorbidities; more episodes and rapid cycling; and greater degrees of treatment resistance during prospective longitudinal follow-up than in the Netherlands and Germany, abbreviated as Europe [12]. The earlier age of onset appears related to an increase in the incidence of genetic vulnerability for bipolar illness and psychiatric illness in general in the parents and grandparents of the patients from the US compared to Europe. In addition, there is also an increase in the incidence of childhood stressors (verbal, physical, and sexual abuse) in the US compared to Europe. Both of these genetic and environmental vulnerabilities are associated with an earlier age of onset of bipolar disorder and a more adverse course of illness in the US. It is noteworthy that the occurrence of verbal abuse alone (without the often accompanying physical or sexual abuse) is sufficient to be associated with the earlier age of onset and a more severe course of illness. As such, family-focused therapy (FFT) or another related family-based psychotherapy is a crucial component to the treatment of the precursors and earliest manifestations of the illness. This kind of therapy in addition to medications is associated with less depression and better outcomes, especially in families with highly expressed emotion [13,14].

Lithium is both less frequently used and started later in the course of illness in the US than in Europe. This could be a contributing factor in addition to the genetic and environmental vulnerabilities to the more adverse course of illness in the US. In addition to the antimanic effects of lithium demonstrated in controlled clinical trials, long-term follow-up data of Geller et al. [15] and Hafemen et al. [16] suggest that the use of lithium in youth with bipolar disorder is associated with a better outcome than with other agents and there is less depression and suicidal ideation when lithium is used compared with anticonvulsants and atypical antipsychotics. Thus, greater use of lithium in both childhood- and adult-onset bipolar disorder may help change the trajectory of illness into a more benign and less disabling form.

## 2. The Unique Assets of Lithium

Lithium not only prevents episodes but also prevents all of the indices of illness progression or what has been referred to as sensitization-like effects or the tendency of the illness to worsen as a function of the number of prior recurrences [8,17]. In addition to greater numbers and severity of episodes, more episodes are associated with greater cognitive impairment and treatment resistance. These sensitization effects are mediated by epigenetic effects based on chemical elements added to DNA and histones and changes in microRNA that alter how readily or not genes are expressed. Many of the epigenetic marks on sperm and ova survive after fertilization and alter vulnerability to illness in the next generation. Thus, in addition to the greater genetic vulnerability in the US compared to Europe, there is additional epigenetic vulnerability from the increases in parental stressors, episodes, and bouts of substance abuse that are also more prevalent in the US [12,17]. In addition, there are three times as much assortative mating (where two parents with mood disorders marry each other) in the US than in Europe, thus leaving more children in the US with a bilineal genetic as well as epigenetic vulnerability for psychiatric illness.

Therefore, given the high risk for childhood-onset bipolar illness in the US (7–9) and its relationship to a more adverse course of illness, extra measures should be taken to attempt to head off the multiple and progressive abnormalities associated with an increasing number of episodes (6, 13). As such, lithium should be given much higher consideration for early use in heading off episodes and illness progression [9,10,11,15,16]. In the case of medical illnesses such as multiple sclerosis (MS) [18] and rheumatoid arthritis (RA) [19], when disease-modifying drugs (DMDs) are used early in the course of these illnesses, they prevent the associated anatomical progression and functional deterioration accompanying these illnesses. Patients with these illnesses understand that delaying appropriate treatment with DMD drugs places them on a trajectory of irredeemable anatomical abnormalities and functional deficits. Given the many attributes of lithium that would appear to merit its designation as a disease-modifying drug (DMD) [5], earlier use may also help head off the neurobiological, anatomical, and functional deterioration that typically accompanies bipolar illness.

Parents with bipolar illness need to know that their children are at high risk for bipolar disorder and its related comorbidities such as ADHD, anxiety disorders, oppositional defiant disorder, and substance abuse [12,20]. Yet childhood-onset illness in the US is poorly recognized and treated. In addition to the diagnosis of early onset bipolar disorder itself, the delay to first treatment is longer in the US than in Europe and this delay is an independent predictor of more depression and a poor outcome in adulthood [21]. As such, parents with bipolar illness can assist clinicians and physicians in the identification and progression of impairing symptoms in their children.

An Institutional Review Board-approved system is available for parents to each week rate the severity of anxiety, depression, ADHD, oppositional behavior, and mania in their child so that the ratings can be printed out and shown to physicians in order to more systematically view illness course and response to treatment [22]. This system can be accessed at www.bipolarnews.org, accessed on 8 August 2024 (click on Child Network). These weekly parental ratings can readily display the range of inadequately treated symptoms in a child and help in the development and assessment of more effective treatment regimens.

Lack of and delayed use of lithium should be viewed from much the same perspective as that in MS or RA. Many of the deficits associated with multiple recurrences of episodes are not readily reversible or reversible at all. This is the case when one considers the many other fundamental assets of lithium in addressing the mechanism underlying the pathophysiology of the illness and illness progression. These deserve to be briefly enumerated here.

Lithium increases gray and white matter volume in the brain, and it is unique in increasing the volume of the hippocampus. It does this by increasing several neuroprotective factors BDNF and Bcl-2 and inhibiting cell death factors such as BAX and P-53 [23]. Thus, describing lithium in popular literature as toxic is a complete misnomer. It possesses the opposite characteristics of increasing cell survival. Lithium increases neurogenesis; it reduces all-cause mortality and extends longevity [24]. These are not trivial outcomes, as patients with bipolar disorder normally lose more than a decade of life expectancy, primarily based on increases in cardiovascular events, such as strokes and heart attacks [25]. Lithium also has the strongest data for preventing suicides which occur at a higher rate in bipolar disorder than in any other psychiatric illness [26].

Lithium increases the length of the end strands of DNA called telomeres, which shorten with depression, stress, and aging and are associated with increases in physical and psychiatric illnesses and comorbidities. Lithium activates the enzyme telomerase which places more DNA on the ends of telomeres [27] and does so in proportion to the duration of time a patient is treated with lithium [28].

Lithium also helps resolve some of the fundamental abnormalities associated with bipolar disorder, such as altered circadian rhythms. In addition, it has immunomodulatory effects and even anti-inflammatory and anti-viral effects [7].

While lithium has these unique properties, it is a too common misconception that if treatment with lithium leads to long-term remission, it is possible to slowly taper the drug and discontinue it. The data indicate that stopping lithium in the face of a good long-term response does not lessen the risk of a high incidence of relapse, about 50% within 5 months off the drug [29]. In a very small percentage of patients, there is an additional risk of not responding as well as they did before (or even at all) when treatment with lithium is re-instituted [30]. The first author knows about a dozen patients who have experienced this grievous outcome, and they all wanted others to know that stopping a treatment that has been working well in the long term, is not without considerable risk. This includes a severe relapse, suicide, or inducing an illness that has progressed to the next stage of lithium non-responsiveness and even refractoriness to treatment with multiple other agents as well.

## 3. Lithium’s Adverse Effects Have Been Generally Over-Emphasized

One of the most widely touted side effects of lithium has been its causing kidney damage that can lead to the necessity of dialysis. The International Group for The Study of Lithium Treated Patients (IGSLI) evaluated lithium’s impact on kidneys in 312 patients treated with lithium for 8–48 years (mean 18 years). It was concluded that long-term lithium treatment was associated with a gradual decline of renal functioning by about 30% more than that due to aging alone. The GFR declined by 0.7%/year of age and 0.9%/year of treatment, both by 19% more among women than men [31]. However, a very large nationwide study in Denmark has revealed that patients treated with lithium are no more likely to develop end-stage renal failure than those treated with anticonvulsants such as valproate or lamotrigine [32]. Moreover, recent changes in the way lithium is administered further decrease the likelihood of renal damage. This includes giving lithium in once-nightly doses, attempting to keep levels in the lower end (0.6–0.8 meq/L) of the therapeutic range (0.6 to 1.1 meq/L), and avoidance of episodes of lithium toxicity.

A more common side effect of lithium on the kidneys is lithium’s ability to block the effects of the antidiuretic hormone vasopressin resulting in increases in urination called diabetes insipidus (DI). If urine amounts become problematic, DI can usually be well managed with the addition of the diuretic amiloride which markedly cuts the urine volume and frequency [33].

One often sees the potentially alarming statement that lithium is toxic to the thyroid. This disparaging and misleading characterization distorts the explanation that lithium can interfere with the production of thyroid hormones T3 and T4 and induce hypothyroidism in some 15 to 20% of patients. However, this deficit in hormone production can readily be alleviated with the replacement of thyroid hormone T4 [33].

Another common side effect of lithium is tremors, but in most cases, these can be avoided by lowering the dose of lithium. If this is not effective, the administration of propranolol at doses of 20–80 mg/day is a potentially effective strategy [33]. In cases where the patient is highly sensitive to lithium tremors, and an adequate dose cannot be achieved, lithium can be supplemented with the dihydropyridine calcium channel blocker nimodipine which has lithium-like effects without its tremor or other side effects [34,35]. Nimodipine specifically blocks the calcium influx gene CACNA1C which has been repeatedly linked to an increase in vulnerability to bipolar disorder and depression.

Weight gain is often erroneously touted as an invariable and problematic side effect of lithium. However, recent large long-term follow-up studies suggest that on average this is not the case [36].

Another widely held belief is that lithium impairs cognition or creativity. On the contrary, there is considerable evidence that over one year compared to placebo lithium in low doses prevents cognitive decline in those with mild cognitive impairment [37] and bipolar patients who use lithium have a lower incidence of dementia in old age than nonusers [38].

There are a variety of other less common potential side effects of lithium, but most can be dealt with by appropriate adjuncts or alternatives.

## 4. Questions That Patients with Bipolar Disorder Can Ask Their Physicians (Especially If They Are Not Already Being Treated with Lithium)

(1) Why am I not on the best medicine for treating and preventing bipolar illness progression?
(2) I have had one manic episode; is it not time to use lithium to head off further episodes and their long-term adverse consequences, including more rapid, severe, and untriggered relapses; cognitive dysfunction; disability; and ultimately treatment nonresponsiveness?
(3) I am on these other medications but still having episodes. Is it not time to add in lithium as it enhances the effectiveness of most other agents?
(4) If you as a physician or nurse practitioner are not well versed in the management of patients on lithium, can you refer me for a second opinion to an expert who is that I can see periodically?
(5) Bipolar illness is largely a progressive illness, is it not time that I used the best preventative medicine for it?

## 5. Some Counterintuitive Statements about Lithium

(1) Lithium is not a toxic drug in its usual doses. It is neuroprotective and reparative [7,39]. It increases the amount of gray matter and white matter in the brain, increases the volume of the hippocampus, and increases longevity.
(2) Since lithium likely qualifies as a disease-modifying drug (DMD), its use should be mandated earlier and more frequently in preventing the progression of bipolar illness [40].
(3) Bipolar illness is one of the psychiatric illnesses with the highest suicide rate, but lithium has the best data for preventing suicide [27]. There are also 10 studies indicating that micro-doses of lithium in drinking water are associated with lower rates of suicide in the general population [41].
(4) Lithium is an essential vitamin/mineral. Without its presence, normal growth and development do not occur. Li+ in lithium carbonate is an ion that has many similarities to the commonly used salt Na+ in sodium chloride, readily crosses cell membranes, and enters most cells in the brain and body. There are a myriad of potential mechanisms of action of lithium, but the ion has the exceptional properties of preventing or reversing most of the severe consequences of bipolar disorder. Calling Li+ the awesome ion or the incredible ion would not appear inappropriate.
(5) Contrary to the even lower utilization of lithium in bipolar disorder in children compared to adults, lithium is highly effective in childhood mania [42], and in long-term follow-up studies, children on lithium have better outcomes, including more periods of remission and less depression and suicide [15,16].
(6) Lithium in low doses prevents deterioration of mild cognitive impairment [37], and in therapeutic doses reduces the incidence of dementia in old age [38].
(7) There is almost no reason not to consider the use of lithium in patients with bipolar disorder. It has an extraordinary range of assets beyond its anti-manic effects (see Table 1 below).
(8) Most investigators now agree that prophylactic treatment of bipolar disorder should begin after a first mania, as two studies have shown that the recurrence of new episodes in the first year is associated with a lack of normalization of the deficits in cognition that occur compared to those with no further episodes.

The multiple assets of lithium beyond its anti-manic effect which were demonstrated in recent years are depicted in Table 1.

## 6. Avenues for Future Research

A key area for future research would be the conduct of randomized controlled clinical trials of lithium in those at high risk for bipolar disorder by a parent with a diagnosis of bipolar disorder and in those with prodromal symptoms meeting criteria for bipolar not otherwise specified and related early manifestations of illness. Randomized clinical trials of lithium compared to other agents in early-onset bipolar disorder would also be of importance. For example, Berk et al. [43] reported that after a first hospitalization for mania, randomization to one year of treatment with lithium was superior to one year of quetiapine on all measures of mood, functioning, and abnormalities on brain imaging.

Clinical predictors of a good response to lithium include those with a BP I diagnosis with distinct episodes of euphoric mania, a lack of anxiety and substance abuse comorbidity, fewer episodes and less rapid cycling, and a positive family history of bipolar disorder and a good response to lithium. Valid neurobiological predictors of a good response to lithium remain to be ascertained and brought into clinical practice. When lithium is insufficient in producing a remission, further research is needed to more definitively assess what are the next best steps. This will be of considerable importance as lithium is able to enhance the efficacy of most other agents, including the mood-stabilizing anticonvulsants (lamotrigine, carbamazepine, and valproate); the dihydropyridine calcium channel blocker nimodipine; and virtually all of the atypical antipsychotics [44,45]. Only a select few AAs have definitive antidepressant effects in patients with bipolar depression. These include quetiapine in adults, lurasidone in children aged 10–17 and in adults, cariprazine, and lumateperone, but not aripiprazole. Lurasidone in both children and adults treated for bipolar depression is of particular interest because of the drug’s good tolerability and weight neutrality and the unusual findings that the drug has the largest effect size in patients who have evidence of inflammation (high level of CRP) at baseline.

Another area for future study would be how to best address the multiple comorbidities that frequently accompany bipolar disorder but are less responsive to lithium including a wide range of anxiety and substance abuse comorbidities, eating disorders, and compulsions such as trichotillomania and nonsuicidal self-injury. These would be important areas for further study, especially since some commonly used supplements and other agents appear to show promising results. One of these N-acetylcysteine (NAC) has shown positive results in stabilizing mood and anxiety, in multiple types of addictions, obsessive compulsive disorder, trichotillomania, and other pathological habits [44,45]. Another is acetyl-L-carnitine which is low in the blood of patients with refractory depression (especially those with early onsets and a history of childhood adversity). It has good effects in depressed patients and in the defeat-stress animal model of depression where it works more rapidly than conventional antidepressants and appears to do so by an epigenetic mechanism of inducing an inhibitory glutamate receptor which decreases glutamate release [44,45]. The role of folate and L-methylfolate also deserves further exploration as enhancers of lithium and other antidepressant effects.

The promising area of further research on lithium is also that of pharmacogenetics, as indicated by the recent paper of Adiukwu et al. [46]. The gene variations that influence response to lithium were studied by the “candidate gene” method and more recently by the genome-wide association studies (GWASs). In 2021, two excellent reviews of the pharmacogenetics of lithium response were published [47,48].

## 7. Conclusions

Patients with multiple sclerosis (MS) and rheumatoid arthritis (RA) who do not use disease-modifying drugs (DMD) early in the course of their illness know they risk incurring irreversible anatomical and functional deficits. In parallel, patients with bipolar illness who do not use lithium which might also be called a DMD, risk incurring irreversible anatomical and functional deficits, including cognitive dysfunction, disability, dementia, and premature loss of life expectancy. However, some caution must be exercised since functional and morphological measurements in MS and RA, even if not perfect, are easier to estimate than behavioral, emotional, or neuromorphological changes in bipolar illness.

Bipolar disorder as it is conventionally treated in the United States is associated with a poor prognosis. Reversing the unwarranted neglect of lithium therapy in a very large segment of the patient population has the chance of making bipolar disorder a much less pernicious illness. The 75th anniversary of the introduction of lithium should be an occasion for the reassessment of the appropriate role of this drug in the treatment of the many adversities of bipolar disorder and reversing the many decades of its underutilization [49].

## Figures and Tables

**Table 1:** Multiple assets of lithium beyond its anti-manic effects that patients should consider.

Variable	Data
Diagnosis	Lithium prevents unipolar and bipolar depression.
Suicide	Lithium prevents suicides in patients and in the general population.
	Lithium prevents suicide in the general population at minuscule doses in the water supply.
Augmentation	Lithium enhances the effects of other mood stabilizers and atypical antipsychotics.
Hippocampus	Lithium increases the volume of the hippocampus.
Gray/white matter	Lithium prevents or reverses cortical gray matter and white matter tract deficits.
Circadian rhythm	Lithium normalizes circadian rhythm abnormalities and has immune-modulating effects.
Immune system	Lithium has immune-modulating effects.
Telomeres	Lithium increases the length of telomeres shortened by episodes, stress, and aging.
Memory	Lithium prevents or slows memory deterioration in mild cognitive impairment over one year.
Dementia	Lithium reduces the incidence of a diagnosis of dementia in old age.
Life expectancy	Lithium prolongs life expectancy and reduces the incidence of all-cause mortality.
All-cause mortality	Lithium reduces the incidence of all-cause mortality
